# Differential Stress-Induced Neuronal Activation Patterns in Mouse Lines Selectively Bred for High, Normal or Low Anxiety

**DOI:** 10.1371/journal.pone.0005346

**Published:** 2009-04-28

**Authors:** Patrik Muigg, Sandra Scheiber, Peter Salchner, Mirjam Bunck, Rainer Landgraf, Nicolas Singewald

**Affiliations:** 1 Department of Pharmacology and Toxicology, Institute of Pharmacy, Center for Molecular Biosciences Innsbruck (CMBI), University of Innsbruck, Innsbruck, Austria; 2 Max Planck Institute of Psychiatry, Department Behavioral Neuroendocrinology, Munich, Germany; University of Wuerzburg, Germany

## Abstract

There is evidence for a disturbed perception and processing of emotional information in pathological anxiety. Using a rat model of trait anxiety generated by selective breeding, we previously revealed differences in challenge-induced neuronal activation in fear/anxiety-related brain areas between high (HAB) and low (LAB) anxiety rats. To confirm whether findings generalize to other species, we used the corresponding HAB/LAB mouse model and investigated c-Fos responses to elevated open arm exposure. Moreover, for the first time we included normal anxiety mice (NAB) for comparison. The results confirm that HAB mice show hyperanxious behavior compared to their LAB counterparts, with NAB mice displaying an intermediate anxiety phenotype. Open arm challenge revealed altered c-Fos response in prefrontal-cortical, limbic and hypothalamic areas in HAB mice as compared to LAB mice, and this was similar to the differences observed previously in the HAB/LAB rat lines. In mice, however, additional differential c-Fos response was observed in subregions of the amygdala, hypothalamus, nucleus accumbens, midbrain and pons. Most of these differences were also seen between HAB and NAB mice, indicating that it is predominately the HAB line showing altered neuronal processing. Hypothalamic hypoactivation detected in LAB versus NAB mice may be associated with their low-anxiety/high-novelty-seeking phenotype. The detection of similarly disturbed activation patterns in a key set of anxiety-related brain areas in two independent models reflecting psychopathological states of trait anxiety confirms the notion that the altered brain activation in HAB animals is indeed characteristic of enhanced (pathological) anxiety, providing information for potential targets of therapeutic intervention.

## Introduction

Pathological fear and anxiety and its physiological expression can be conceptualized as representing a continuum, ranging from persistent anxiety not attributable to specific factors to exaggerated responses to a perceived threat or a bias towards interpreting ambiguous situations as threatening [1]. Dysfunctional excitability in neurons of the anxiety/fear circuitry is speculated to be a common abnormality in anxiety disorders [1]. While it is well established that certain brain areas are involved in the perception and processing of acute fear (for review, see [2]–[4]), much less is known about the regional nature of changes in proposed anxiety circuitries (for review, see [5]) predisposing individuals to be hyperanxious. One approach toward obtaining a better understanding of these mechanisms is to use psychogenetically selected rodent lines, developed from a common foundation population that exhibit consistent and robust differences in the selection criterion. Using this strategy, various rat lines have been generated that differ in certain aspects of emotionality including anxiety, such as the Maudsley Reactive and Nonreactive strain, Roman high and low avoidance rat lines, Tsukuba strains, and high/low anxiety-related behavior (HAB/LAB) rat lines (for review, see [6]–[9]). To our knowledge, corresponding mouse models selectively bred for extremes in anxiety are not available, with one exception (see below).

In HAB/LAB rats, systematic immediate early gene expression studies succeeded in mapping differences in neuronal activity patterns underlying behavioral responses to a variety of aversive situations [10]–[14]. So far, no such information is available in mice. Recently, HAB and LAB mouse lines have been established by selective and bidirectional breeding for high (HAB) and low (LAB) anxiety-related behavior measured on the elevated plus-maze (EPM)[15], [16]. Compared to LAB mice, HAB mice were more anxious and showed increased risk assessment behavior in a number of tests, including the EPM test, open-arm exposure test, light/dark avoidance test and ultrasound vocalization test. Moreover, unselected CD1 “normal” anxiety-related behavior (NAB; for definition, see Material and Methods) mice, as well as HAB/LAB F1 intercrosses, displayed intermediate behavioral scores in most of the tasks performed [16].

In the present study, we aimed for the first time to investigate in mice whether genetically determined differences in anxiety-related behavior as well as risk assessment would be reflected by differential stress-induced c-Fos expression as a reliable marker of neuronal activation in key brain areas of anxiety circuitries previously described in rats (for review, see [14]).

Placement of HAB, NAB and LAB mice on an open arm (OA) of the EPM was chosen as a mild anxiety-based stressor. The investigation of c-Fos in NAB mice provides an additional advantage, as the intermediate phenotype of these animals may act as a reference of changes in brain activity, suitable to determine whether HAB or LAB mice reveal neuronal alterations. By confirming similarly affected neuronal populations in an additional model of a different species, the general significance of the previous findings would be strengthened, suggesting the difference in trait anxiety presumably being the cause. At the same time, this cross-species replication would provide further information as to possible effects of genetic drift, giving rise to genetic differences that are unrelated to the selected phenotype. Differences in c-Fos activation, in other words, could reflect both the selection pressure and drift-related phenomena.

## Materials and Methods

### Animals

#### Ethics statement

The study described here was designed to minimize animal suffering and number of animals used, and was approved by the local Ethical Committees on Animal Care and Use.

All animals tested were bred in the animal facilities of the Max Plank Institute of Psychiatry in Munich (Germany) as described previously [16]. Briefly, >250 animals from >25 litters of outbred Swiss CD1 mice purchased from Charles River were used as a starting point for selective and bidirectional breeding for anxiety-related behavior on the EPM at the age of seven weeks, with at least six families routinely maintained within each selected line. Males and females that spent either the least or most time on the open arms of the EPM were mated to establish the HAB and LAB mouse lines. This intra-line approach was chosen to make sure that the HAB/LAB lines show a maximum divergence in the selected trait, while maintaining a high degree of similarity in non-selected traits. All experiments in the present study were carried out on inbred adult male HAB (n = 14), LAB (n = 13) and NAB (n = 13) mice (22–25g body weight; 13–14 weeks of age). The animals were routinely tested at an age of 7 weeks in Munich with HAB and LAB mice spending less than 10% and more than 50% of their time, respectively, on the open arms of the EPM. This was also the selection criterion. NAB mice are bred for “normal” (i.e. intermediate) anxiety-related behavior. They were selected from a group of CD1 mice maintained in the laboratory while HAB and LAB lines were being selected. As >80% of CD1 mice spent between 25% and 35% of their time on the open arms of the EPM, this range was chosen for the selection of NAB mice without any overlapping either with HAB or with LAB animals. While CD1 mice in the parental generation were used as NAB controls by Krömer *et al*. [16] and Kessler *et al*. [15], we then decided to start inbreeding them in parallel with HAB and LAB mice to further reduce variables that are unrelated to anxiety, such as slight differences in body weight between outbred vs. inbred animals. In the present study, HAB and LAB mice of generations 18–22 and NAB mice of generations 1–3 were used. Importantly, in a wide variety of tests and parameters, the intermediate scores of (bred) NAB, (purchased) CD1, and HAB/LAB F1 controls were found to be similar if not identical ([16]; unpublished results). We generated six independent families within the HAB, LAB and NAB lines using a within-family selection design.

In Innsbruck, HAB, NAB and LAB animals were housed under standard laboratory conditions (12:12 h light/dark cycle with lights on at 7:00; 21°C; 50% humidity; pelleted food; and water *ad libitum*) for 6 weeks in groups of 3–4 litter mates per cage. At least 24 h before the experiment, animals were taken in their home cages to the experimental rooms for habituation. The behavioral test was carried out during the light phase of the cycle (between 8:30 and 12:30 a.m.).

### OA exposure

Mice (HAB: n = 9, LAB: n = 8, NAB: n = 8) were placed in the middle of the OA (50×5 cm) of an EPM (facing the proximal compartment). The maze was elevated 73 cm above the floor and illuminated by a light intensity of 100 lux. Access to the neutral zone and the closed arms of the maze was prevented by a bar that made it impossible for the mouse to leave the OA. The arm was thoroughly cleaned with water before the introduction of each mouse and divided into a distal, a middle and a proximal zone. The behavior of the mice during the 5-min testing period was analyzed by an automatic videotracking system (Videomot 2.0, TSE, Bad Homburg, Germany). In addition, the test session was videotaped for later analysis of the head-dip behavior by an experienced observer using the Eventlog 1.0 (EMCO Software). The behavioral parameters scored included the number of entries into the distal zone of the OA, the time spent in the distal zone of the OA, total distance traveled, number of head dips below the surface of the OA, the time spent head dipping and the latency until the first head dip. Immediately after behavioral testing, animals were returned to their home cages. Animals assigned to the control (basal) group (n = 5 for all three lines) were not exposed to the OA and were taken directly from their home cages for further analysis.

### c-Fos immunohistochemistry

**Figure 1:**
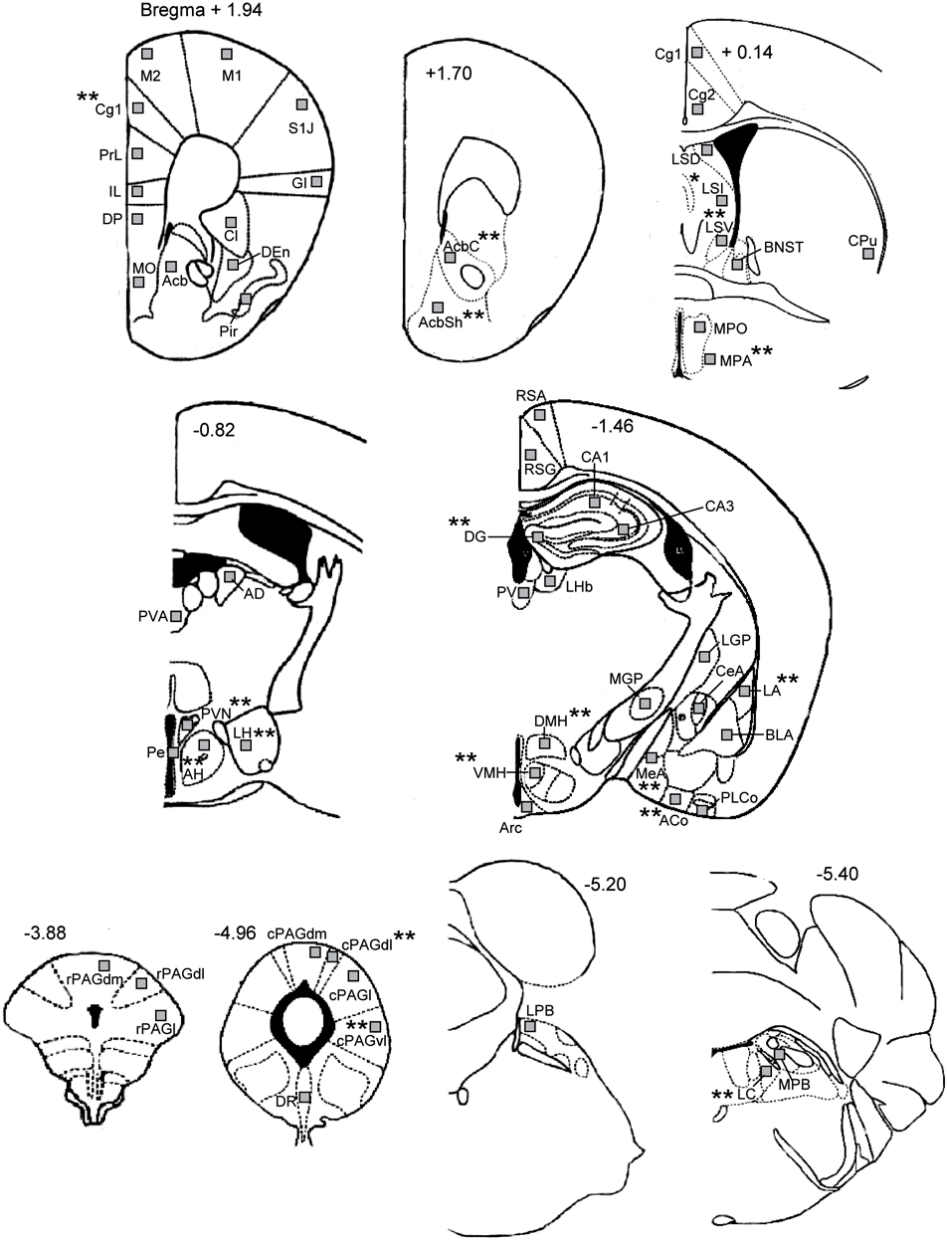
Schematic diagram showing the 59 areas in which c-Fos expression was quantified. Levels are based on the atlas of Franklin and Paxinos (1997). Squares indicate the placement of grids for counting of c-Fos positive cells. Asterisks indicate the regions in which HAB mice showed changes in OA-induced c-Fos expression as compared to LABs. AcB, nucleus (n.) accumbens; AcBc, n. accumbens core; AcBsh, n. accumbens shell; ACo, anterior cortical n. of the amygdala; AD, anterodorsal thalamic n.; AH, anterior hypothalamic area; Arc, arcuate hypothalamic nucleus; BlA, basolateral n. of the amygdala; BNST, bed n. of the stria terminalis; CA1, CA1 field of the hippocampus; CA3, CA3 field of the hippocampus; CeA, central n. of the amygdala; Cg 1, cingulate ctx (area1); Cg 2, cingulate ctx (area2); Cl, Claustrum; CPu, caudate putamen; cPAGdl, caudal dorsolateral periaqueductal gray; cPAGdm, caudal dorsomedial periaqueductal gray; cPAGl, caudal lateral periaqueductal gray; cPAGvl, caudal ventrolateral periaqueductal gray; DEn, Endopiriform ctx, dorsal; DG, dentate gyrus; DMH, dorsomedial hypothalamic n.; DP, dorsal peduncular nucleus; DR, dorsal raphe n.; GI, granular insular ctx; IL, infralimbic ctx; LA, lateral n. of the amygdala; LC, locus coeruleus; LGP, lateral globus pallidus; LH, lateral hypothalamic area; LHb, lateral habenular n.; LPB, lateral parabrachial n.; LSD, lateral septal n. (dorsal); LSI, lateral septal n. (intermediate); LSV, lateral septal n. (ventral); M1, primary motor ctx; M2, secondary motor ctx; MeA, medial amygdala; MGP, medial globus pallidus; MO, medial orbital cortex; MPA, medial preoptic area; MPO, medial preoptic n.; MPB, medial parabrachial n.; PE, periventricular n; Pir, piriform ctx; PLCo, posterolateral cortical n. of the amygdala; PrL, prelimbic ctx; PV, paraventricular thalamic n.; PVA, paraventricular thalamic n. (anterior); PVN, paraventricular hypothalamic n.; rPAGdl, rostral dorsolateral periaqueductal gray; rPAGdm, rostral dorsomedial periaqueductal gray; rPAGl, rostral lateral periaqueductal gray; RSA, retrosplenial agranular ctx; RSG, retrosplenial granular ctx; S1J, primary somatosensory cortex, jaw region; VMH, ventromedial hypothalamic n.

The maximum level of c-Fos protein can be detected between 1 and 3 h following an acute challenge, then it gradually disappears from the cell nucleus [17]–[19]. Therefore, 2 h after the onset of the OA exposure test, animals were deeply anesthetized with an overdose of sodium pentobarbital (200 mg/kg) and transcardially perfused with 100 ml of 0.9% saline followed by 100 mL of 4% paraformaldehyde in 0.1 mol/L phosphate buffered solution (PBS, pH 7.4). Mice not exposed to the test paradigm were treated identically immediately after removal from their cages in the experimental room. Brains were then removed and postfixed at 4°C overnight in 4% paraformaldehyde in PBS. Coronal sections (100 µm) were cut with a Vibratome (Ted-Pella, Inc., Redding, CA, USA) and collected in Immunobuffer. C-Fos immunoreactivity was performed in three runs for i) the rostral (from Bregma +1.94mm to +0.14mm), ii) the middle (from Bregma −0.82mm to −1.46mm) and iii) the caudal part (from Bregma −3.88mm to −5.40mm) of the mouse brain. Within a given run, sections of all groups were processed simultaneously in order to avoid batch effects. The sections were processed as described previously [20]. Briefly, sections were incubated for 72 h in a polyclonal rabbit anti-c-Fos primary antibody (sc-52, Santa Cruz Biotechnology, Santa Cruz, CA, USA) diluted (1∶20000) in immunobuffer (pH 7.4). The sections were then rinsed and placed in a biotinylated goat anti-rabbit secondary antibody (1∶200, Vector Laboratories, Burlingame, CA, USA) for 24 h. An avidin-biotin-horseradish peroxidase procedure (Vectastatin ABC Kit, Vector laboratories, Burlingame, USA) with 3,3′-diaminobenzidine (DAB, Sigma, Germany) as chromogen was used to visualize c-Fos positive cells. The incubation time with the DAB-solution was 10 min for all sections. The chromogen reaction was initiated by the addition of the H_2_O_2_ solution (0.004%) and terminated after 7 min (colour change to brown) by adding Tris buffer (50mM). The staining procedure generally yielded low background staining and differential staining intensities of c-Fos positive cells. A cell was considered as c-Fos-labeled (c-Fos positive), if the brown-black DAB-stained nucleus was unambiguously darker than background staining, which included all cells from low to high intensities of staining. The lighting of the microscope was optimized for the best visibility of c-Fos labeled cells and kept constant for all sections. The c-Fos quantification was performed at different levels of the brain in 59 different structures, which (amongst others) are known to show stress-induced increase in c-Fos expression [13], [21], [22] . Many of these regions have been implicated in the anxiety circuitry (for review, see [5], [23], [24]). The anatomical localization of c-Fos-positive cells was aided by use of adjacent Nissl stained sections and the illustrations in a stereotaxic atlas [25]. The anterior-posterior levels of sections included for detailed analysis and associated structures are shown in Figure 1. The number of c-Fos-positive cells was quantified bilaterally in a tissue area of 100 µm×100 µm. This was performed for the whole experiment by one and the same well-experienced observer, who was blind to the experimental groups. Generally, cell counting was performed maximally for 3 h per day in the time period from 9 to 12 a.m.

### Statistical Analysis

Statistical analysis using the Kolmogorov-Smirnov test and Shapiro-Wilk test (Software: Statistica 7.1, Statsoft Inc.,USA) revealed parametric (normal) distribution for the behavioral data and the c-Fos data for most brain regions. Therefore, overall statistical analysis of behavioral data was performed using the 1-way ANOVA followed by Fischer LSD *post hoc* analysis. The number of c-Fos positive cells was analysed by using the 2-way ANOVA followed by (if there was a significant *line x stress* interaction in ANOVA) Fischer LSD *post hoc* analysis to detect statistically significant differences between the groups. Correlations between the parameter distal arm entries and the number of c-Fos cells were performed using the Spearman's rank correlation coefficient test. The level of significance was set at P<0.05. All values were expressed as mean±SEM.

## Results

### OA behavior

**Figure 2:**
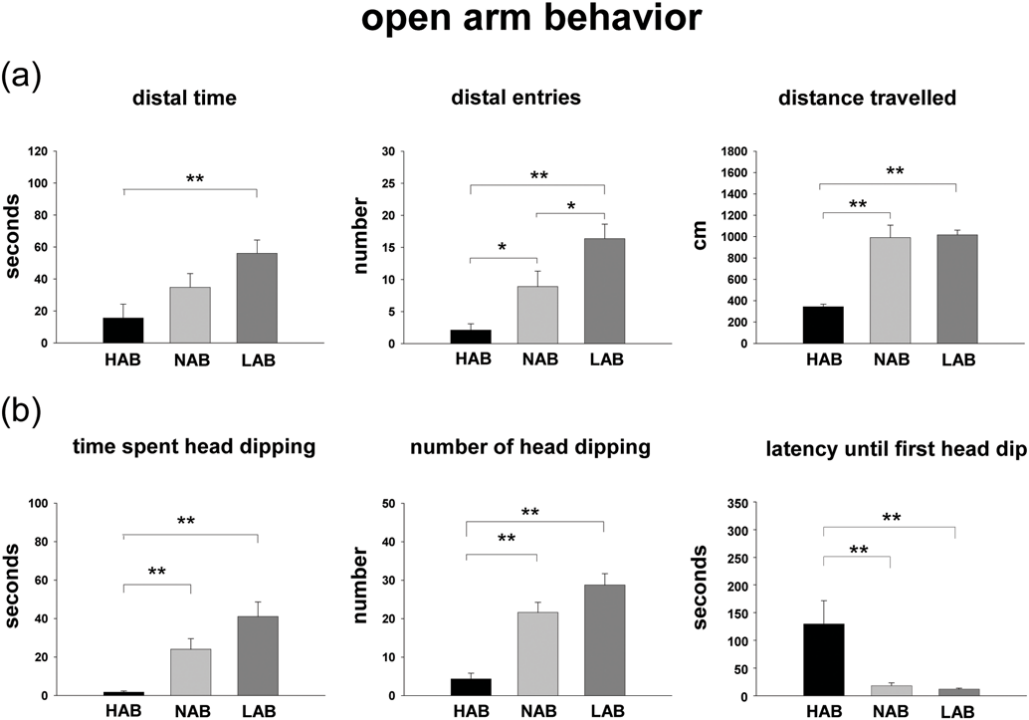
Behavioral parameters of HAB, NAB and LAB mice measured in the 5-min exposure to the OA. (a) Time spent in distal zone, entries into distal zone, total distance traveled. (b) Head-dip behavior. Values are expressed as mean±SEM. HAB: n = 9, NAB: n = 8, LAB: n = 8; * p<0.05, ** p<0.01.

ANOVA analysis revealed significant differences between the three mouse lines for the parameters “distal time” (F(2,24) = 5.759, P = 0.010), “distal entries” (F(2,24) = 13.880, P<0.001), “distance traveled” (F(2,24) = 48.895, P<0.001), “time spent head dipping” (F(2,24) = 14.810, P<0.001), “number of head dips” (F(2,24) = 28.715, P<0.001) and “latency until first head dip” (F(2,24) = 6.413, P = 0.006). During OA exposure, the Fischer exact revealed that the time spent in the distal zone and the number of entries into the distal zone of the OA were significantly lower in HAB than in LAB mice (see Figure 2). NAB animals displayed intermediate anxiety-related behavior, although the difference for the parameter “time spent head dipping” failed to reach statistical significance from HAB and LAB lines (Figure 2a). Risk assessment behavior as indicated by head dips below the surface of the OA [26] was higher in LAB and NAB mice compared with their HAB counterparts. In HAB animals the number of head dips and the duration of head dipping were significantly lower, while the latency until the first head dip was significantly higher as compared with NAB and LAB mice. Head-dip behavior did not differ between NAB and LAB mice (Figure 2b).

### Line Differences in OA-induced c-Fos expression

An overview of the 59 brain areas in which c-Fos expression was quantified is given in Figure 1. Mean numbers±SEM of cells expressing c-Fos in these brain regions are shown in Table 1.

**Table 1:** Overview of c-Fos expression following open arm (OA) exposure in HAB, NAB and LAB mice.

	BASAL			OPEN ARM			2-way ANOVA
	HAB	NAB	LAB	HAB	NAB	LAB	(line x stress)
Brain regions (brain level)							
**Cortical areas**							
Prelimbic cortex ##	2.4±0.5	2.7±0.5	1.8±0.3	14.2±1.2	11.4±1.4	11.2±1.1	F(2,24) = 1.300, P = 0.297
Infralimbic cortex ##	1.5±0.7	1.2±0.1	1.0±0.4	10.2±1.0	9.3±1.6	7.7±1.1	F(2,24) = 1.533, P = 0.243
Cingulate cortex 1 (+1,94) ##	1.3±0.4	1.7±0.3	1.7±0.9	6.8±0.6	9.4±1.3	9.1±0.5	**F(2,24) = 5.958, P = 0.010**
Cingulate cortex 1 (+0,14) ##	1.5±0.4	2.0±0.8	2.0±0.8	17.9±1.6	16.6±2.5	19.7±2.1	F(2,24) = 2.314, P = 0.128
Cingulate cortex 2 ##	1.4±0.8	1.6±0.7	1.5±0.2	15.9±1.3	14.0±1.6	16.6±1.6	F(2,24) = 0.480, P = 0.626
Piriform cortex ##	3.8±0.8	5.8±0.7	5.1±1.5	18.2±1.8	14.8±2.8	15.8±1.3	F(2,24) = 1.346, P = 0.285
Primary motor cortex ##	1.7±0.6	2.5±0.7	2.8±0.8	6.0±0.8	5.8±1.4	7.1±1.0	F(2,24) = 1.478, P = 0.255
Secondary motor cortex ##	2.1±0.7	3.0±1.2	2.7±0.7	10:0±0.7	8.6±1.6	10.2±1.1	F(2,24) = 11.278, P = 0.281
Endopiriform cortex, dorsal ##	0.5±0.2	0.5±0.2	0.7±0.3	1.5±0.3	3.4±1.0	2.2±0.6	F(2,24) = 2.219, P = 0.138
Orbital cortex, medial	4.7±1.0	4.6±0.8	5.3±0.8	4.1±0.8	6.7±0.8	5.9±2.5	F(2,24) = 0.115, P = 0.892
Peduncular nucleus, dorsal ##	1.4±0.4	2.0±0.5	1.1±0.3	7.9±0.8	7.8±2.1	7.6±1.3	F(2,24) = 3.276, P = 0.061
Primary somatosensory ctx #	1.4±0.8	1.0±0.5	1.2±0.5	2.8±0.4	1.8±0.4	3.8±1.0	F(2,24) = 0.899, P = 0.424
Granular insular cortex	1.2±0.6	1.2±0.6	2.3±0.4	2.5±0.2	1.6±0.4	3.8±1.2	F(2,24) = 0.523, P = 0.602
Retrosplenial agranular cortex ##	3.6±0.9	4.5±1.6	6.1±2.7	27.9±4.8	22.4±2.8	22.9±2.5	F(2,24) = 0.749, P = 0.162
Retrosplenial granular cortex ##	1.5±0.7	2.2±0.5	1.6±0.5	16.9±2.1	15.6±2.6	20.2±1.8	F(2,24) = 1.259, P = 0.308
**Basalganglia**							
Caudate putamen	0.2±0.1	0.3±0.1	0.2±0.1	0.1±0.1	0.4±0.2	0.2±0.1	F(2,24) = 0.043, P = 0.958
Lateral globus pallidus	0.2±0.1	0.2±0.2	0.3±0.1	0.6±0.2	0.8±0.3	0.8±0.1	F(2,24) = 0.862, P = 0.439
Medial globus pallidus	0.2±0.1	0.2±0.1	1.3±0.9	0.4±0.1	0.7±0.4	0.7±0.1	F(2,24) = 0.321, P = 0.729
Nucleus accumbens ##	0.8±0.3	0.7±0.4	0.6±0.3	7.7±0.7	6.8±1.4	6.1±0.5	F(2,24) = 3.249, P = 0.062
Nucleus accumbens, core ##	0.9±0.2	0.5±0.2	0.8±0.4	8.9±0.8	6.2±1.1	6.0±0.8	**F(2,24) = 9.120, P = 0.002**
Nucleus accumbens, shell ##	0.4±0.1	0.6±0.3	0.8±0.2	4.3±0.4	2.8±0.5	2.9±0.5	**F(2,24) = 7.004, P = 0.006**
Claustrum ##	2.0±0.5	2.4±0.5	2.5±0.4	11.5±0.8	10.1±2.2	9.3±0.7	F(2,24) = 1.821, P = 0.190
**Striatal sections**							
Lateral septum, intermediate ##	0.8±0.3	0.8±0.2	1.4±1.7	12.8±1.3	9.1±2.1	9.6±1.2	**F(2,24) = 9.221, P = 0.002**
Lateral septum, ventral ##	6.5±0.6	7.9±0.4	6.3±1.7	17.8±0.7	10.0±1.5	10.3±0.9	**F(2,24) = 3.565, P = 0.049**
Lateral septum, dorsal #	0.5±0.2	1.5±0.5	0.4±0.2	3.4±0.6	2.6±0.9	4.6±0.7	F(2,24) = 3.311, P = 0.060
Bed n. of stria terminalis ##	1.3±0.4	2.0±0.3	2.5±1.0	8.1±1.3	6.4±1.4	8.1±1.1	F(2,24) = 3.497, P = 0.052
**Thalamus**							
Paraventricular thalamic n. ##	9.3±0.9	10.2±0.6	8.0±1.5	42.6±3.7	35.0±2.8	33.8±2.6	F(2,24) = 0.836, P = 0.450
Lateral habenular nucleus ##	5.5±2.8	6.7±1.7	8.3±0.8	26.6±5.4	26.2±5.5	30.3±5.4	F(2,24) = 0.183, P = 0.834
Paraventricular n., anterior ##	4.5±0.7	5.3±0.6	5.3±0.6	11.7±1.3	13.3±1.8	10.3±1.2	F(2,24) = 0.071, P = 0.932
Anterodorsal thalamic nucleus	0.7±0.3	0.6±0.5	0.3±0.1	0.3±0.1	0.2±0.2	0.2±0.1	F(2,24) = 0.390, P = 0.683
**Hypothalamus**							
Paraventricular hypothalamic n. ##	2.3±1.1	1.2±0.5	1.3±0.3	12.2±1.3	10.5±1.6	3.8±1.0	**F(2,24) = 12.115, P<0.001**
Periventricular hypothalamic n. ##	2.4±0.2	2.4±0.7	3.7±1.2	7.6±0.7	6.6±1.9	7.6±0.8	F(2,24) = 0.039, P = 0.962
Medial preoptic nucleus ##	2.0±0.4	2.4±0.5	3.3±0.5	13.9±1.0	12.9±1.8	13.1±2.2	F(2,24) = 1.048, P = 0.371
Medial preoptic area ##	4.7±0.3	5.5±1.0	4.5±0.4	16.3±0.7	12.1±0.6	8.3±0.6	**F(2,24) = 27.728, P<0.001**
Lateral hypothalamic area ##	2.0±0.3	2.7±0.3	3.6±0.7	11.4±0.8	4.9±0.2	4.6±0.5	**F(2,24) = 23.446, P<0.001**
Dorsomedial hypothalamic n. ##	4.8±1.5	3.0±0.7	5.1±1.2	22.2±2.4	13.9±0.3	12.3±1.6	**F(2,24) = 10.068, P = 0.001**
Arcuate hypothalamic nucleus ##	2.6±0.5	2.4±0.6	1.7±0.3	8.6±1.4	7.3±2.0	9.7±1.7	F(2,24) = 2.297, P = 0.129
Ventromedial hypothalamic n. ##	1.3±0.3	1.9±0.1	2.9±0.8	15.5±1.4	5.6±0.6	7.3±1.4	**F(2,24) = 12.934, P<0.001**
Anterior hypothalamic nucleus	6.8±1.0	9.7±2.1	6.0±0.6	13.2±0.6	10.6±0.5	6.8±0.5	**F(2,24) = 4.556, P = 0.025**
**Amygdala**							
Central n. of the amygdala	2.2±0.3	1.5±0.7	2.4±0.8	3.4±0.5	2.9±0.6	3.5±0.9	F(2,24) = 0.256, P = 0.777
Medial n. of the amygdala ##	3.2±0.1	2.9±0.6	3.3±1.1	14.7±0.6	11.8±1.0	11.0±0.9	**F(2,24) = 4.284, P = 0.048**
Lateral n. of the amygdala ##	0.6±0.1	0.9±0.3	0.4±0.2	4.9±0.4	3.5±0.3	2.9±0.5	**F(2,24) = 5.901, P = 0.011**
Basolateral n. of the amygdala ##	1.3±0.7	1.5±0.4	1.8±0.4	7.0±0.9	6.5±0.9	6.9±0.6	F(2,24) = 1.352, P = 0.284
Posterolateral cortical amy. ##	1.4±0.2	1.9±0.7	1.2±0.5	8.2±1.3	10.0±0.7	7.3±2.3	F(2,24) = 0.413, P = 0.668
Anterior cortical amygdala ##	2.5±0.3	3.1±0.7	3.0±0.5	9.8±0.6	6.4±0.4	6.2±1.0	**F(2,24) = 5.800, P = 0.011**
	**BASAL**			**OPEN ARM**			**2-way ANOVA**
	**HAB**	**NAB**	**LAB**	**HAB**	**NAB**	**LAB**	(line x stress)
**Brain regions**							
**Hippocampus**							
Dentate gyrus ##	33.0±4.5	32.4±4.5	35.4±3.9	64.5±6.5	110.1±13.6	127.6±10.8	**F(2,24) = 5.552, P = 0.013**
CA1 field ##	27.4±2.8	32.3±3.6	31.4±3.4	61.5±8.5	58.8±7.7	57.0±8.5	F(2,24) = 0.412, P = 0.668
CA3 field ##	18.8±1.9	17.4±2.7	23.9±3.6	59.7±6.9	63.9±4.2	58.2±4.6	F(2,24) = 1.498, P = 0.250
**Midbrain/pons**							
PAG rostral, dorsomedial ##	7.6±1.8	6.2±0.6	5.9±0.5	16.7±1.6	12.3±2.	14.1±1.6	F(2,24) = 2.048, P = 0.158
PAG rostral, dorsolateral ##	4.0±0.8	3.9±0.2	3.0±0.2	12.7±0.5	9.3±0.6	8.4±0.6	F(2,24) = 2.797, P = 0.088
PAG rostral, lateral ##	5.0±0.5	5.3±0.8	5.3±1.2	24.9±1.4	20.2±2.6	24.4±1.3	F(2,24) = 3.518, P = 0.051
PAG caudal, dorsomedial ##	1.5±0.4	1.8±0.4	2.3±0.4	6.4±0.7	6.4±2.1	5.4±0.7	
PAG caudal, ventrolateral ##	5.0±0.4	6.7±0.9	4.8±0.4	19.3±0.8	16.4±0.8	15.6±1.5	**F(2,24) = 3.587, P = 0.049**
PAG caudal, lateral ##	2.0±0.3	3.1±1.0	3.0±0.7	17.9±1.1	14.1±3.8	17.6±1.0	F(2,24) = 2.721, P = 0.093
PAG caudal, dorsolateral ##	2.5±0.7	3.7±0.7	2.7±0.7	13.2±1.3	6.7±1.5	6.9±1.5	**F(2,24) = 11.522, P = 0.001**
Dorsal raphe nucleus ##	1.0±0.2	1.8±0.3	1.5±0.3	7.8±1.4	6.3±1.1	6.8±2.2	F(2,24) = 1.014, P = 0.383
Lateral parabrachial nucleus ##	2.7±0.9	3.8±1.5	4.1±1.3	6.6±1.1	5.6±0.9	8.8±1.3	F(2,24) = 1.474, P = 0.255
Medial parabrachial nucleus	1.1±0.4	0.6±0.2	1.1±0.2	0.4±0.2	0.9±0.3	1.1±0.3	F(2,24) = 0.966, P = 0.399
Locus coeruleus ##	3.2±0.3	3.6±1.1	2.4±0.4	15.1±1.2	10.3±0.6	9.6±0.4	**F(2,24) = 11.983,P<0.001**

Values are numbers of c-Fos positive cells/0.01 mm^2^. (total number of c-Fos positive cells was quantified in the CA1 and CA3 region and the dentate gyrus of the hippocampus). 2-way ANOVA analysis results for *line x stress* interaction are given in the right column (brain areas showing significant interaction are shown in bold). 2-way ANOVA analysis results for the factor *stress* are indicated by # P<0.05, ## P<0.01 basal versus OA stress groups; basal groups: n = 5, OA-groups: n = 8–9;

#### Basal c-Fos expression

In mice of the basal groups which were not exposed to the OA, the number of cells expressing c-Fos was low in most areas examined. Moderate numbers of c-Fos-positive cells were, however, detected in some cortical, thalamic, and hypothalamic areas. No differences in basal c-Fos expression were observed among HAB, NAB and LAB mice (Table 1).

#### c-Fos expression after OA exposure

OA exposure induced c-Fos expression in a variety of brain areas, with moderate to pronounced increases in areas involved in stress responses (see Table 1, 2-way ANOVA analysis for the factor *stress*), including different cortical areas, limbic areas such as subregions of the amygdala, the bed nucleus of the stria terminalis, areas of the hippocampal formation, the lateral septum, the nucleus accumbens, as well as the thalamic and hypothalamic nuclei, parts of the periaqueductal gray (PAG) and diverse brainstem nuclei. Only in a few areas did OA stress fail to induce a significant increase in c-Fos expression, including in the medial orbital and granular insular cortices, the medial and lateral globus pallidus, the caudate putamen, the anterodorsal thalamic nucleus, the central nucleus of the amygdala and the medial parabrachial nucleus.

2-way ANOVA analysis revealed a significant line x stress interaction for the number of c-Fos positive cells in 18 out of 59 brain areas investigated, including the cingulate cortex, the nucleus accumbens (core, shell), the lateral septum (ventral, intermediate), the paraventricular hypothalamic nucleus (PVN), the medial preoptic area, the lateral hypothalamic area, the dorsomedial hypothalamic nucleus, the ventromedial hypothalamic nucleus, the anterior hypothalamic nucleus, the medial nucleus of the amygdala, the lateral nucleus of the amygdala, the anterior cortical amygdala, the dentate gyrus, the caudal periaqueductal gray (ventrolateral, dorsolateral) and the locus coeruleus (LC). For details of statistics (F and P values) see Table 1.

**Figure 3:**
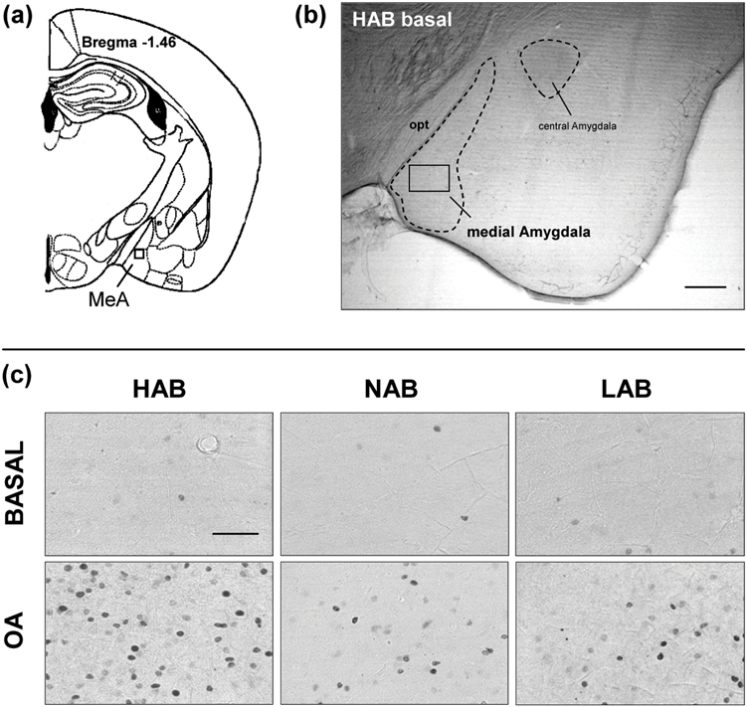
Representative microphotographs of c-Fos immunoreactivity in the amygdala. (a) Schematic diagram, based on the atlas of Franklin and Paxinos (1997), showing the amygdala at the level of −1.46 (Bregma). The square indicates the placement of grids for counting of c-Fos-positive cells in the medial nucleus of the amygdala (MeA). (b) Low magnification overview of the amygdala (−1.46) of a HAB mouse under basal conditions; Scale bar = 500 µm; (c) High magnification, bright field photomicrographs of representative sections matched for comparable rostrocaudal levels showing the distribution of c-Fos expression within the medial nucleus of the amygdala in HAB, NAB and LAB mice under basal conditions and after OA exposure. Scale bar = 100 µm;

**Figure 4:**
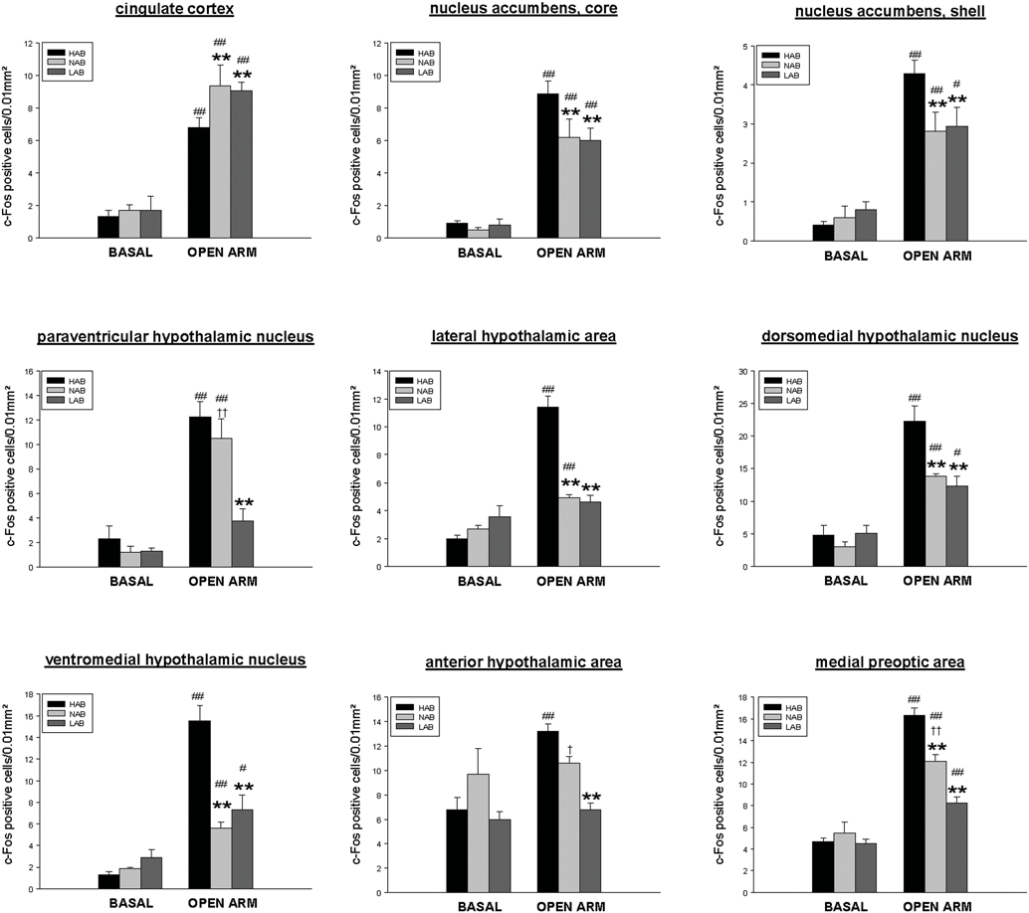
Quantitative analysis of c-Fos immunoreactivity in HAB, NAB and LAB mice under basal conditions and after OA exposure. Depicted are those areas (cortical, accumbal and hypothalamic areas), for which the Fischer LSD *post hoc* test revealed statistically significant differences in OA-stress-induced c-Fos response in HAB, NAB and LAB mice. Each column indicates the mean±SEM number of c-Fos positive cells in a tissue area of 0.01mm^2^ (total c-Fos expression was quantified in the dentate gyrus). Basal groups: n = 5, OA-exposure: HAB: n = 9, NAB: n = 8, LAB: n = 8; *p<0.05, **p<0.01 vs HAB OA-group; # p<0.05, ## p<0.01 vs corresponding basal group; + p<0.05, ++ p<0.01 vs LAB OA-group;

**Figure 5:**
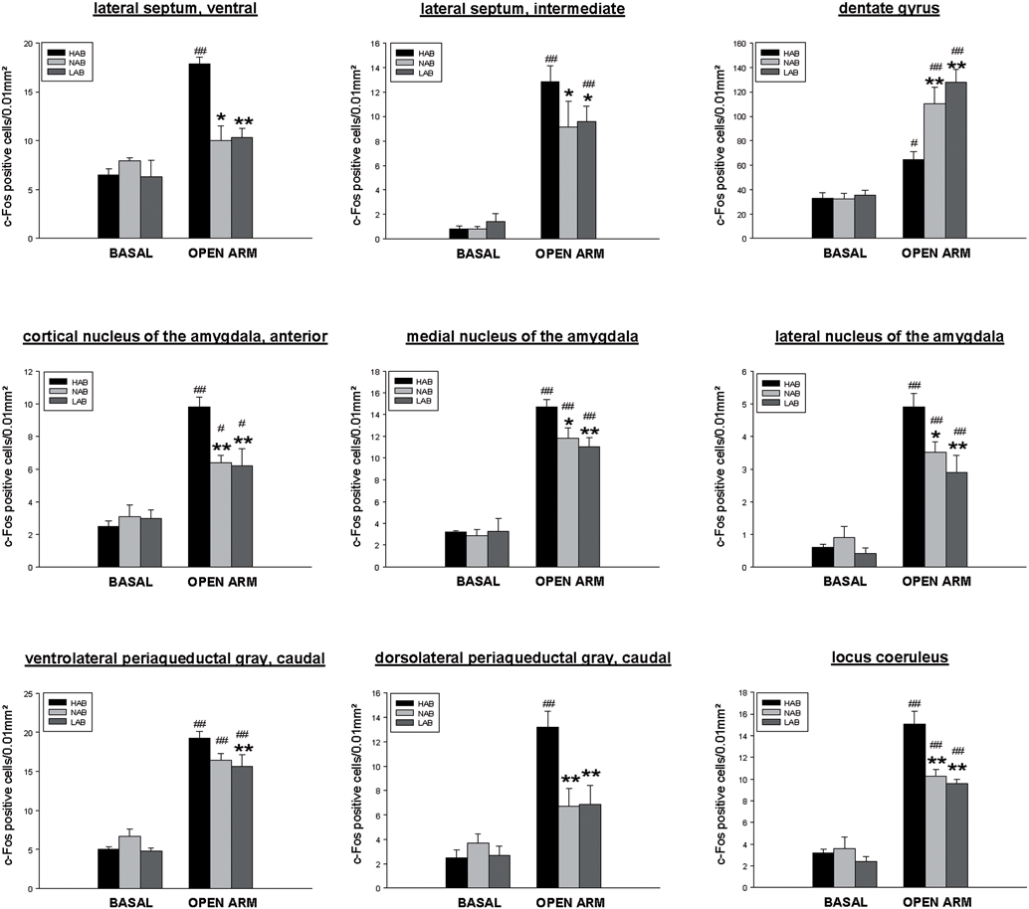
Quantitative analysis of c-Fos immunoreactivity in HAB, NAB and LAB mice under basal conditions and after OA exposure. Depicted are those areas (septal, hippocampal, amygdalar and hind brain areas) for which the Fischer LSD *post hoc* test revealed statistically significant differences in OA-stress-induced c-Fos response in HAB, NAB and LAB mice. Each column indicates the mean±SEM number of c-Fos positive cells in a tissue area of 0.01mm^2^ (total c-Fos expression was quantified in the dentate gyrus). Basal groups: n = 5, OA-exposure: HAB: n = 9, NAB: n = 8, LAB: n = 8; *p<0.05, **p<0.01 vs HAB OA-group; # p<0.05, ## p<0.01 vs corresponding basal group; + p<0.05, ++ p<0.01 vs LAB OA-group;

In all 18 brain areas, *post hoc* analysis revealed a differential OA-induced c-Fos response between HAB and LAB mice. The c-Fos response to OA exposure was increased in HAB as compared with LAB mice, in 16 areas, including the shell and the core region of the nucleus accumbens, the ventral and intermediate part of the lateral septum, the PVN, the lateral and anterior hypothalamic area, the dorsomedial and ventromedial hypothalamic nucleus, the medial preoptic area, the medial, lateral and anterior cortical amygdala, the caudal PAG (ventrolateral, dorsolateral) and the LC (Figure 3, Figure 4, Figure 5). Conversely, a lower number of c-Fos positive cells in HAB than in LAB animals was found in 2 areas, the cingulate cortex and the dentate gyrus of the hippocampus (Figure 4, Figure 5).

In 15 of these 18 areas a similar difference in the neuronal activation pattern was seen, when NAB were compared with HAB mice. The exception was the PVN, the anterior hypothalamic area and the caudal periaqueductal gray (ventrolateral), where the statistically significant difference in the c-Fos response was lost when HAB were compared with NAB animals. Thus, the neuronal activation response of NAB and LAB mice was very similar in most of these areas, but LAB mice displayed a lower c-Fos response compared with NABs in the PVN, the anterior hypothalamic area and the medial preoptic area (Figure 4). In all other brain areas, OA-induced c-Fos expression in NAB mice did not differ from HAB or LAB mice. Since we used a number of comparisons (59 for each brain area), it should be noted that there is the theoretical possibility of false positive results, although the probability of this is very low.

#### Correlation analysis between distal arm entries and c-Fos expression

Correlation analysis between OA *distal arm entries* and *c-Fos expression* after OA exposure was performed for the 18 brain areas where HAB and LAB mice showed significant differences in c-Fos response (see above). The Spearman test revealed significant negative correlation for the PVN, the lateral hypothalamic area, the dorsomedial hypothalamic nucleus, the ventromedial hypothalamic nucleus, the anterior hypothalamic area, the medial preoptic area, the lateral septum (ventral), the medial nucleus of the amygdala, the lateral nucleus of the amygdala and the LC, indicating that higher anxiety behavior (fewer distal entries) is correlated with enhanced c-Fos response in these areas. Along these lines, a positive correlation was found for the dentate gyrus. None of the other brain regions correlated significantly with the *distal arm entries* during OA exposure. Details of the correlation analysis (including R and P values) are given in Table 2.

**Table 2:** Correlation analysis of *distal arm entries* and *c-Fos responses* in brain areas where HAB, NAB and LAB mice showed significant differences in OA stress-induced c-Fos response.

	DISTAL ARM ENTRIES	
	Spearman	
BRAIN AREAS	R	P
Cingulate cortex	0.298	0.148
Nucleus accumbens, core	−0.121	0.566
Nucleus accumbens, shell	−0.136	0.518
**Paraventricular hypothalamic nucleus**	**−0.533**	**0.006**
**Lateral hypothalamic area**	**−0.706**	**<0.001**
**Dorsomedial hypothalamic nucleus**	**−0.551**	**0.033**
**Ventromedial hypothalamic nucleus**	**−0.616**	**0.015**
**Anterior hypothalamic area**	**−0.721**	**<0.001**
**Medial preoptic area**	**−0.733**	**<0.001**
**Lateral septum, ventral**	**−0,523**	**0.007**
Lateral septum, intermediate	−0.347	0.089
**Dentate gyrus**	**0.722**	**0.002**
Cortical nucleus of the amygdala, anterior	−0.495	0.061
**Medial nucleus of the amygdala**	**−0.653**	**0.008**
**Lateral nucleus of the amygdala**	**−0.603**	**0.017**
Ventrolateral periaqueductal gray, caudal	−0.298	0.148
Dorsolateral periaqueductal gray, caudal	−0.375	0.065
**Locus coeruleus**	**−0.529**	**0.005**

R and P values revealed by the Spearman test are presented for each brain region.

## Discussion

This study demonstrates that HAB mice display clear signs of a hyperanxious phenotype in the OA exposure test as compared to LAB mice, while NAB animals show intermediate anxiety-related behavior. It furthermore confirms in large part the OA-induced brain activity pattern previously found in the corresponding HAB/LAB rat model (see [13]). The striking new finding of the present HAB/LAB mouse study is that this mild anxiogenic stimulus invoked differences in neuronal activation patterns in additional brain areas, including subregions of the amygdala, the hypothalamus, the nucleus accumbens, the midbrain and the pons, and that the OA-induced neuronal activation profile in NAB mice resembled in large part that of LAB mice. Therefore it is suggested that it is predominantly the HAB line that shows altered processing of mild anxiety-provoking stimuli, thus substantiating the search for correlates of anxiety-related phenomena particularly in this line [7].

### Line differences in OA behavior

The behavioral results of the present study confirm previous observations demonstrating that HAB mice are more anxious [16] and show higher risk assessment behavior than LAB mice in the OA exposure test. This is consistent with the observations made in HAB and LAB rats during OA exposure [13], [27]. NAB mice essentially displayed an intermediate anxiety-like phenotype compared with the extreme lines, although their head dip behavior was similar to that of the LAB line.

Interestingly, in the present study we noted reduced “total distance traveled” of HAB animals compared with both LAB and NAB mice. While the distance traveled, which is rather low due to the small size of the OA, may be considered to reflect locomotor activity, decreased activity is also considered to be a form of anxiety in which a “behavioral inhibition system” is activated [3]. Therefore, immobility (behavioral inhibition) *per se* is proposed to be an indirect sign of intense anxiety/fear states in rodents. This is also reflected by the fact that HAB animals [16] and rodents in general showing high levels of anxiety- and depression-like behavior display reduced general locomotor activity [28]–[31] and vice versa [32], [33]. Moreover, while traveling similar distances, NAB mice also clearly differed from LAB mice in anxiety-related parameters, further supporting the critical role of anxiety (rather than locomotor effects) in the behavioral divergence among HAB, NAB and LAB animals as observed in the OA exposure test.

### Differential c-Fos expression in response to OA exposure in HAB and LAB mice

The development of anxiety-related symptoms is closely related to stress coping [14], [34]–[38]. Stress-induced neuronal activation is thought to delineate neuronal stress circuitries in rats and mice [22], [39]–[43]. Exposure to the OA of an EPM is considered as a mild stressor inducing higher fear/anxiety than exposure to the closed arm [44]–[46]. Indeed, the present study revealed that mice stressed by OA exposure, compared to non-stressed (basal) mice, showed enhanced c-Fos expression in widespread brain regions related to fear/anxiety.

While the OA-induced c-Fos response in HAB compared to LAB rats was *increased* in 5 out of 8 differentially modulated brain areas (the lateral septum [ventral part], the PVN [parvocellular part], the medial preoptic area, the anterior hypothalamic area and the lateral hypothalamic area), *attenuated* c-Fos response was found in the cingulate cortex as well as in the dentate gyrus and the CA3 region of the hippocampus [13]. Remarkably, in the present study we were able to confirm these findings in the corresponding mouse model. In all but one of these regions, HAB and LAB mice displayed the same differences in neuronal activation after exposure to the OA. Only in the CA3 region of the hippocampus did the mouse lines display no differences. As extensively discussed by Salome *et al*. [13], disturbances in these brain areas and associated systems may contribute to behavioral and neuroendocrine responses typical of high trait anxiety.

The present study in mice, however, identified a number of additional brain areas showing increased c-Fos response in HAB versus LAB mice not seen in the rat experiment. These included various limbic areas (subregions of the amygdala, the dorsomedial and ventromedial hypothalamic nucleus, the nucleus accumbens [core and shell], the lateral septum) as well as PAG subregions and the LC. Supporting the relationship between anxiety-like behavior and c-Fos response in particular brain areas, we were able to demonstrate a significant correlation between distal arm entries and c-Fos response in specific amygdaloid and hypothalamic regions, in the lateral septum ventral, in the LC, and in the dentate gyrus. In the rat model, line differences in c-Fos response in these more widespread regions were observed only when stronger challenges were used, such as social defeat, airjet, FG-7142 injection or forced swimming (for review, see [14]). Hence, the HAB/LAB mouse model may be particularly sensitive in reflecting phenotype-specific neuronal activation. However, another explanation could be that the additional differences not common to the two species are not related to differences in trait anxiety. Future studies using anxiolytic drugs and additional challenges not related to the EPM may help to clarify this issue. While the HAB/NAB/LAB lines are of the same CD1 background, we cannot completely rule out the possibility of a different time course in c-Fos responses between the lines. This may represent a potential limitation of the present study. Nevertheless this seems unlikely given that all three lines are of the same CD1 background.

A central finding of the present study is the evidence of a hypersensitive amygdala and a hyposensitive prefrontal cortex (cingulate cortex) in HAB mice. These areas are homologous with important fear-/anxiety-related regions of the human brain [47]–[49], thus resembling the situation in stressor-exposed post-traumatic stress disorder patients with poor top-down control of the amygdala by structures such as the medial prefrontal cortex [50], [51]. In the present study, OA exposure stress enhanced c-Fos expression in the cingulate cortex in all three lines, but the maximum level of activation was reduced in HAB mice compared with LAB and NAB mice. This effect was not very pronounced. However, since cingulate cortex hypoactivation was also found in HAB rats after exposure to various stressors (for review, see [14]), we believe that this response might be a general feature of HAB rats and HAB mice mediating high anxiety levels during stress exposure. Given that the amygdala is a central relay station transferring fear-/anxiety-related information to other brain areas (such as the PAG, the brain stem, and the hypothalamus) for the processing, expression and integration of anxiety-related emotions (e.g. [52]–[54]), it is likely that hyperactivation in the amygdala is involved in the mediation of the increased anxiety-related behavior of HAB mice. The association of fear- and anxiety-related phenomena has recently been highlighted [55]–[58].

The PAG is well known to integrate limbic and emotional inputs with a repertoire of behavioral and autonomic responses [59]–[61], and the LC is also well established as being related to anxiety disorders [34], [62]; in addition, the HPA axis and sympatoadrenal system [62]–[64] produce physiological and behavioral responses to stressful stimuli (for review, see [65]). Thus, hyperactivation of these areas as observed in HAB mice may be a general feature of highly anxious rodents (see also [14]).

### Activation patterns in NAB compared to HAB and LAB mice

To resemble human studies, in which differences in neuronal activity between anxiety patients and healthy subjects are assessed, it is of relevance to include an additional group with “normal” anxiety in animal studies. Thus, for the first time, we here aimed also to map immediate early gene expression in NAB mice and to compare the c-Fos response with that in HAB and LAB mice. In 15 out of the identified 18 key brain areas showing differences in the neuronal activation pattern between HAB and LAB animals, similar differences were seen between HAB and NAB mice. Hence, with a few exceptions (see below), the neuronal activation responses of NAB and LAB animals were largely similar, indicating that in particular the increased anxiety-related phenotype of HAB mice may be associated with altered neuronal processing within specific brain areas.

LAB compared to NAB mice showed differential OA-induced c-Fos responses in some hypothalamic sites, namely the PVN, the anterior hypothalamic area and the medial preoptic area. This activation pattern may be associated with the non-anxiety phenotype of LAB vs NAB mice, and/or with enhanced novelty seeking of these animals (see [66]–[69]). Since, however, not much information is available concerning brain areas critically involved in extreme non-anxiety, such a conclusion remains speculative at present.

In our intra-line approach, selection pressure was exerted on anxiety-related behavior only, while a high degree of similarity was maintained in non-selected traits. This, however, does not necessarily mean that any difference detected between HAB and LAB animals, including that in regional c-Fos expression, is causally related to anxiety or anxiety-linked phenomena. Confounds include, for example, random genetic drift across time, giving rise to genetic differences that are unrelated to the selected phenotype. While drift-related risk cannot be entirely avoided, we tried to reduce it by: (i) running independent families within HAB, NAB and LAB lines [16]; (ii) replicating key findings of c-Fos expression in both mice and rats, using similar inbreeding protocols (this study and [13]); and (iii) showing that, in a pharmacological validation approach, paroxetine treatment attenuated both c-Fos expression and depression-like behaviour [11], further confirming an association between neuronal excitability and the behavioral phenotype beyond genetic drift. Future efforts will focus on testing further how strongly c-Fos and anxiety-related behavior are functionally related, including short-term selection and F2 panel associations. Only convergent information from multiple approaches will give rise to a more objective assessment of drift-related compared to other risks.

In conclusion, the data presented here demonstrate that differential c-Fos responses to an unpleasant emotional challenge are found in specific limbic, cortical, hypothalamic and hindbrain areas of HAB vs LAB mice. Remarkably, the key areas differentially activated after OA exposure in the HAB vs. LAB rat lines [13] could be confirmed in this corresponding mouse model, supporting the notion that the altered brain activation pattern in HAB animals may be a generalized feature, being indeed characteristic of enhanced trait anxiety. Similar brain areas were found to display altered activation processing also in anxiety disorder patients (see references in [14]), underlining the translational value of the present findings. The c-Fos response pattern in NAB mice, which displayed intermediate behavioral scores, was similar to that of LAB mice, both showing clear-cut differences to that of HAB animals, suggesting that it is mainly the HAB mouse line which may show altered neuronal activation at least upon OA exposure. This activation pattern typical of the high anxiety-related phenotype, however, may either indicate exaggerated activation of pathways mediating anxiety or represent dysfunctional adaptive responses which normally serve to suppress anxiety.
